# Salvianolic Acid B Significantly Suppresses the Migration of Melanoma Cells via Direct Interaction with β-Actin

**DOI:** 10.3390/molecules29040906

**Published:** 2024-02-19

**Authors:** Ying Zhang, Wenjuan Zhai, Minqi Fan, Jinjun Wu, Caiyan Wang

**Affiliations:** Joint International Research Laboratory of Translational Cancer Research of Chinese Medicines of the Ministry of Education of the People’s Republic of China, Guangdong Provincial Key Laboratory of Translational Cancer Research of Chinese Medicines, International Institute for Translational Chinese Medicine, Guangzhou University of Chinese Medicine, Guangzhou 510006, China; 20212110034@stu.gzucm.edu.cn (Y.Z.); 20201110594@stu.gzucm.edu.cn (W.Z.); 20211110091@stu.gzucm.edu.cn (M.F.)

**Keywords:** Salvianolic acid B (SAB), melanoma, migration, β-actin

## Abstract

Melanoma is the most aggressive and difficult to treat of all skin cancers. Despite advances in the treatment of melanoma, the prognosis for melanoma patients remains poor, and the recurrence rate remains high. There is substantial evidence that Chinese herbals effectively prevent and treat melanoma. The bioactive ingredient Salvianolic acid B (SAB) found in *Salvia miltiorrhiza*, a well-known Chinese herbal with various biological functions, exhibits inhibitory activity against various cancers. A375 and mouse B16 cell lines were used to evaluate the main targets and mechanisms of SAB in inhibiting melanoma migration. Online bioinformatics analysis, Western blotting, immunofluorescence, molecular fishing, dot blot, and molecular docking assays were carried out to clarify the potential molecular mechanism. We found that SAB prevents the migration and invasion of melanoma cells by inhibiting the epithelial–mesenchymal transition (EMT) process of melanoma cells. As well as interacting directly with the N-terminal domain of β-actin, SAB enhanced its compactness and stability, thereby inhibiting the migration of cells. Taken together, SAB could significantly suppress the migration of melanoma cells via direct binding with β-actin, suggesting that SAB could be a helpful supplement that may enhance chemotherapeutic outcomes and benefit melanoma patients.

## 1. Introduction

Melanoma is one of the deadliest cancers, with a high malignant degree and metastasis [1,2]. Despite representing only 1% of all skin malignancies, melanoma is the most common cause of mortality from skin cancer [3]. Most melanoma cases occur in Asian populations in the extremities, while others occur in the eye, nasopharynx, intestinal, and anal mucosa. Metastasis is a common characteristic of melanoma [4,5]. The 5-year survival rate of primary melanoma is 99%, while that of metastatic melanoma is only 27% [6]. Despite advances in the diagnosis and treatment of melanoma with advanced therapies such as targeted therapy and immunotherapy, the prognosis is still poor, and the recurrence rate is still high [7,8]. Alternative therapeutics with pronounced effectiveness against melanoma are necessary.

β-Actin, which is a highly conserved cytoskeletal structural protein related to cell growth and cell migration [9,10,11], has been regarded as an endogenous housekeeping gene in cells and tissues [12,13]. It is known that tumor growth and metastasis are closely related to the cytoskeleton, which regulates the adhesion and movement of tumor cells [14,15]. Studies have shown that the polymerization, localization, cytoskeleton formation, and overexpression of β-actin may promote the movement and metastasis of colorectal adenocarcinoma cells [16,17,18]. In addition, the different distribution of β-actin in the perinuclear region and the leading edge of cells can affect the polarity and plasticity of tumor cell movement, thereby regulating tumor metastasis [19,20,21]. However, the specific relationship between β-actin and the migration of melanoma cells remains unclear. Therefore, this current study aimed to investigate the role of β-actin involved in melanoma migration.

The epithelial–mesenchymal transition (EMT) process is associated with cancer metastasis and invasion. During EMT, the loss of adhesion of epithelial cells can also lead to changes in the mesenchymal phenotype [22]. EMT is characterized by downregulation of the epithelial cell markers cytokeratin and E-cadherin, and upregulation of the mesenchymal cell markers N-cadherin, vimentin, and fibronectin [23]. Changes in the expression of these epithelial cell and mesenchymal cell markers reduce adhesion between neighboring epithelial cells, and also increase cell motility and remodeling, ultimately leading to tumor cell metastasis and invasion [24]. In a variety of cancers, the loss of E-cadherin is often associated with increased expression of the mesenchymal cadherin N-cadherin and is necessary for tumor cells to acquire aggressiveness.

Nowadays, traditional Chinese medicines and their active components play more and more important roles in the prevention and treatment of various cancers [25,26,27]. Salvianolic acid B (SAB), a water-soluble active component extracted from the roots and rhizomes of herbal medicine *Salvia miltiorrhiza Bunge*, which is a traditional herbal remedy for removing blood stasis and enhancing blood circulation [28,29,30]. SAB is a well-known phenolic acid compound, possesses various effects. Previous studies have shown that SAB has inhibitory effects against a variety of cancers. For example, SAB could inhibit EMT and apoptosis of gastric cancer cells through AKT/mTOR and reduce drug resistance [31,32,33,34,35]. By increasing apoptosis and decreasing oxidative stress, inflammation, and angiogenesis, SAB may slow the growth of breast cancer cells [36]. SAB inhibited non-small cell lung cancer A549 cells by inactivating MAPK and SMAD2/3 signaling pathways [37,38]. In addition, SAB could reverse multi drug resistance in nude mice carrying human colon cancer stem cells [39]. However, the inhibitory effects of SAB against melanoma and the molecular mechanism have yet to be fully elucidated. Therefore, this study first analyzed the potential target genes in melanoma in the databases. We further conducted biological experiments to evaluate the inhibition of SAB against melanoma cells. Last, we measured the affinity between SAB and β-actin, which might provide the clues for the molecular mechanism. Our study could provide new ideas for the treatment of melanoma prone to metastasis and lay the experimental foundation for the wide application of SAB in the prevention and treatment of melanoma in clinical practice. 

## 2. Results

### 2.1. β-Actin Protein Is Highly Expressed in Skin Cancer

**Figure 1:**
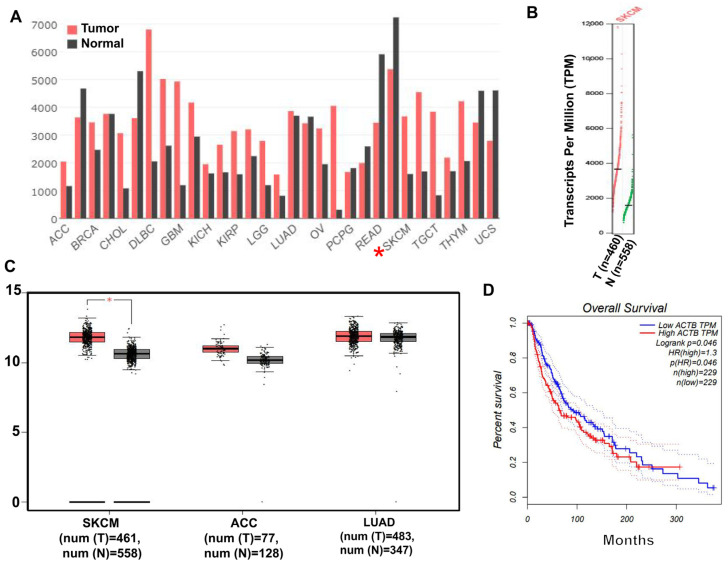
β-Actin protein levels were significantly higher in the SKCM patients than in the normal group. (**A**) Comparison of β-actin expression in normal tissues and cancer tissues, * Stands for SKCM. (**B**) Comparison of actin transcription in normal and SKCM tissues. (**C**) Expression of β-actin in SKCM, ACC, and LUAD tumors, * *p* < 0.05. (**D**) Survival curve analysis of SKCM cancer patients with different β-actin levels.

To investigate the association of β-actin with skin diseases such as melanoma, we analyzed the expression of β-actin in normal tissues and skin tumor tissues through The Cancer Genome Atlas (TCGA) database. Analysis of the data showed that β-actin levels in skin cancer (SKCM) tissues were significantly higher than those in normal tissues (Figure 1A,B). Melanoma is a kind of skin cancer with low incidence, early metastasis, and high malignancy, resulting in higher mortality. Therefore, the expression levels of β-actin in tumor tissues and normal tissues were analyzed by TCGA. We found that the expression of β-actin in the tumor group was higher than that in the normal group (Figure 1C). Finally, we analyzed the relationship between high expression of β-actin and survival rate and found that high expression of actin in SKCM resulted in a decreased survival rate (Figure 1D).

### 2.2. β-Actin Is the Target of Salvia miltiorrhiza for the Treatment of Melanoma through Network Pharmacology Analysis

**Figure 2:**
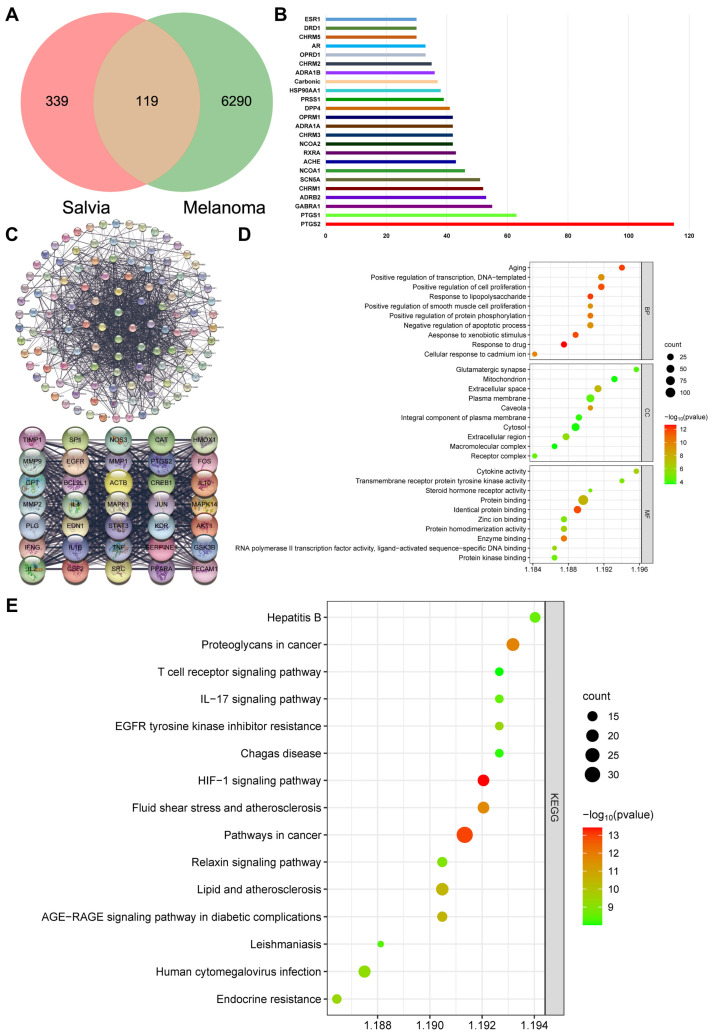
β-Actin is the target of *Salvia miltiorrhiza* for the treatment of melanoma through network pharmacology analysis. (**A**) Venn diagram summarizing the differential and common targets of *Salvia miltiorrhiza* and melanoma. (**B**) The first 20 of 119 targets of *Salvia miltiorrhiza* against melanoma indicated that *Salvia miltiorrhiza* can regulate cell growth and development to be anti-melanoma. (**C**) The process of topological screening for the protein–protein interaction (PPI) network. ACTB (β-actin), also known as β-actin, was located in the center, suggesting that it was the most critical target of *salvia miltiorrhiza* for anti-melanoma. (**D**) GO enrichment analysis of the 119 key targets. The top significant (the cutoff criterion for a significant difference was *p* < 0.05) GO categories are shown. (**E**) KEGG enrichment analysis of the 119 key targets. The top 15 KEGG pathway enrichments are displayed, and only terms with FDR < 0.05 were selected for analysis.

Network pharmacology analysis was conducted to elucidate whether β-actin could be the key target of *Salvia miltiorrhiza* for the treatment of melanoma. Here, 458 targets of active ingredients of *Salvia miltiorrhiza* and 6409 potential therapeutic targets of melanoma were obtained by searching the GeneCards and DisGeNET databases for related research reports after removing the duplicate values. Then, 119 targets that matched the related targets of *Salvia miltiorrhiza* and melanoma were collected as the potential targets of *Salvia miltiorrhiza* against melanoma (Figure 2A). Subsequently, we counted these targets and found that they were related to cell growth and development processes, including cellular oxidative stress and programmed cell death, indicating that *Salvia miltiorrhiza* could regulate cell growth and development processes to resist melanoma (Figure 2B). The protein–protein topological network (PPI network) of those 119 targets was established in the database String to understand the relationship between targets and the course of melanoma (Figure 2C). Then, Cytoscape was adopted for visualizing and integrating those PPI network-involved topological parameters for those 35 screened key targets. We found that the crucial biotargets of *Salvia miltiorrhiza* against melanoma include ACTB, also known as β-actin. β-Actin was in the center of the PPI network, suggesting that it was the most critical target of *salvia miltiorrhiza* for anti-melanoma. To further reveal the potential mechanism of the *salvia miltiorrhiza* against melanoma, GO functional categories and KEGG enrichment analysis of the 37 key targets were performed using the Bioconductor package and the DAVID database (*p* < 0.05), and 533 biological processes (BPs) and 147 signaling pathways were obtained. The top 10 significant (cutoff criterion with a significant difference of *p* < 0.05) GO categories are shown in Figure 2D. The figure showed that the key targets, including β-actin, were mainly involved in the regulation of tumor growth. The top 15 KEGG pathway enrichments were displayed based on the threshold of FDR less than 0.05 (Figure 2E). The figure showed that the key targets were mainly involved in cellular functions and pathways such as cell kinase regulation, cell proliferation, cancer pathways, and polysaccharide regulation of cancer. β-actin was the most critical target of *Salvia miltiorrhiza* against melanoma, proving that β-actin could regulate cell growth and development to have a great effect on anti-melanoma. Based on the above network pharmacology analysis data, we determined that *Salvia miltiorrhiza* could target β-actin to regulate cell growth and development for the treatment of melanoma.

### 2.3. SAB Significantly Inhibited the Proliferation of HaCaT, A375, and B16 Cells

**Figure 3:**
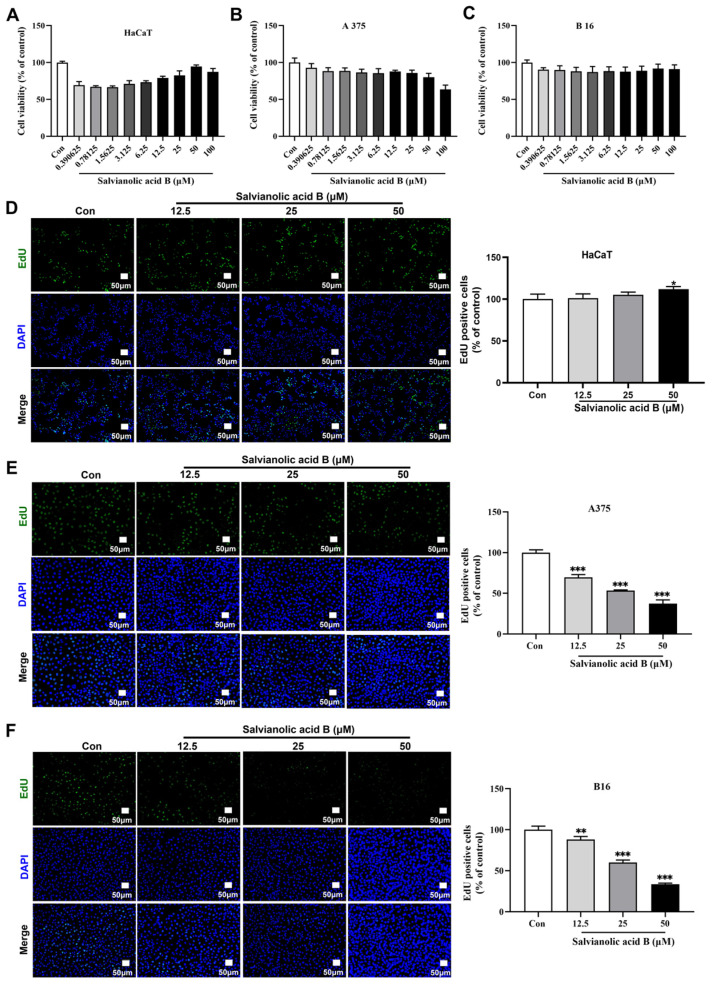
Effects of SAB on the growth and proliferation of HaCaT, A375, and B16 cells. Cytotoxic effects of SAB (0.390625–100 μM, 24 h) on HaCaT (**A**), A375 (**B**), and B16 (**C**) cells were evaluated by using an MTT assay. Effects of SAB (12.5, 25, and 50 μM, 24 h) on the proliferation of HaCaT (**D**), A375 (**E**), and B16 (**F**) cells were evaluated using an EdU kit (scale bar: 50 μm). The number of EdU-positive cells in each group was expressed as a proportion of control cells. The statistics were displayed as the mean ± SD (*n* = 3). In comparison to the control group, * *p* < 0.05, ** *p* < 0.01, and *** *p* < 0.001.

The MTT assay was used to assess SAB’s cytotoxicity toward HaCaT, A375, and B16 cells. After SAB incubation of HaCaT cells at concentrations ranging from 0.390655 to 100 μM for 24 h, we found an interesting result: a low concentration of SAB has a certain inhibitory effect on the viability of HaCaT, but with an increase in concentration of the SAB, the viability of HaCaT cells improved (Figure 3A). SAB incubation of A375 and B16 cells at concentrations ranging from 0.390655 to 100 μM for 24 h did not significantly reduce the viability of the cells (Figure 3B,C). The EdU assay was further conducted to determine the impact of SAB on cell proliferation. After incubation of HaCaT cells with 12.5, 25, and 50 μM SAB for 24 h, it was found that SAB concentrations of 50 μM promoted the proliferation of HaCaT cells (Figure 3D). Incubation of A375 cells with SAB at 12.5, 25, or 50 μM for 24 h markedly decreased the proliferation rate in a dose-dependent manner by 30.21% ± 3.24%, 46.66% ± 0.79%, and 62.54% ± 4.42%, respectively (Figure 3E, *p* < 0.001). These results suggested that the concentration of SAB between 12.5 and 50 μM has a relative proliferative effect on normal skin cells but an inhibitory effect on melanoma cells. In addition, the same treatment significantly and dose-dependently decreased the proliferation rate at B16 cells by 11.75% ± 3.58%, 39.95% ± 3.07%, and 66.44% ± 1.35%, respectively (Figure 3F, *p* < 0.001 or *p* < 0.001).

### 2.4. SAB Significantly Suppressed the Migration of A375 and B16 Cells

**Figure 4:**
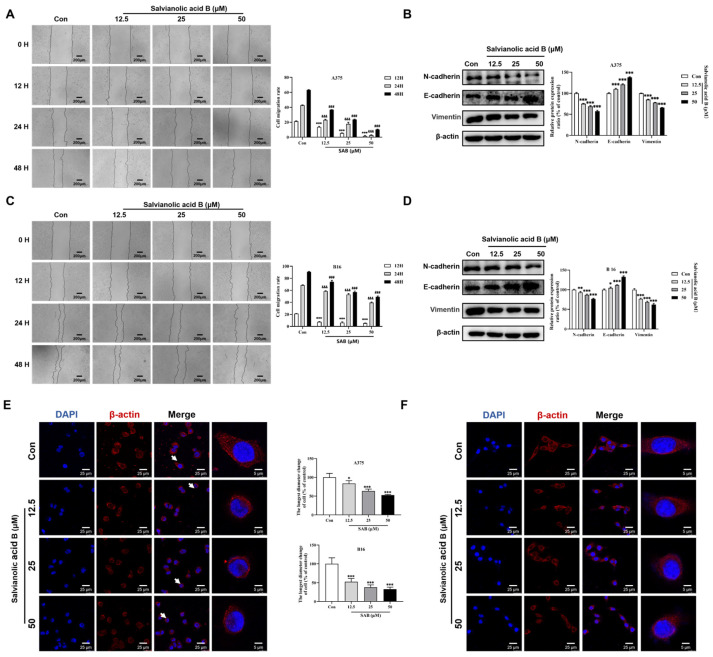
Effects of SAB on the migration of A375 and B16 cells. An approach to studying wound healing was used to study cell migration. A375 (**A**) and B16 (**C**) cells were pretreated with SAB at 12.5, 25, and 50 μM, respectively. Representative photomicrographs (scale bar: 200 μm) of the initial and final wounds were taken after 12, 24, and 48 h at a magnification of 100 times. A375 (**B**) and B16 (**D**) cells were treated with SAB at 12.5, 25, and 50 μM for 24 h. Western blot analysis was used to assess the expression of E-cadherin, N-cadherin, and vimentin. The proportion of control was used to express the protein expression data. The data were presented as mean ± SD (*n* = 3). * *p* < 0.05, ** *p* < 0.01, *** *p* < 0.001, ^&&&^ *p*< 0.001, and ^###^ *p* < 0.001 compared with the 12, 24, or 48 h-treated groups, respectively. A375 (**E**) and B16 (**F**) cells were treated with SAB at 12.5, 25, and 50 μM for 24 h. The white arrows represent pericellular morphology. Representative confocal images (scale bar: 25 μm) of cells double-stained for β-actin (red) and DAPI (blue).

Wound healing assay was performed to study the SAB-mediated inhibition of migration in A375 and B16 cells. As shown in Figure 4A, treatment of A375 cells with SAB at 12.5, 25, 50 μM for 12 h significantly inhibited the cell migration ratio by 36.38% ± 4.15%, 74.01% ± 1.15%, and 92.66% ± 3.66%, respectively (*p* < 0.001). SAB treatment for 24 h significantly inhibited the cell migration ratio by 45.71% ± 1.94%, 58.06% ± 4.97%, and 93.46% ± 0.99%, respectively (*p* < 0.001). In addition, SAB treatment for 48 h markedly inhibited the cell migration ratio by 41.98% ± 0.35%, 62.45% ± 0.70%, and 83.64% ± 0.72%, respectively (*p* < 0.001). As shown in Figure 4C, the similar results were observed in B16 cells. The cell migration ratios were significantly and dose-dependently inhibited by SAB incubation under the same conditions (*p* < 0.001).

The protein levels of E-cadherin, N-cadherin, and vimentin were determined using Western blotting analysis. As shown in Figure 4B, compared to the control cells, the expression of N-cadherin and vimentin in A375 cells exposed to the SAB was significantly decreased, while the expression of E-cadherin was significantly increased (*p* < 0.001). As displayed in Figure 4D, the identical treatment of B16 cells also considerably upregulated the expression of E-cadherin (*p* < 0.001) while significantly downregulating the expression of N-cadherin and vimentin in a dose-dependent manner (*p* < 0.01 or *p* < 0.001).

Then, we studied the impact of SAB on the morphology of A375 and B16 cells by immunofluorescence assay. By staining with β-actin in A375 (Figure 4E) and B16 (Figure 4F) cells, it was found that under the same treatment of SAB, the cells became pyknotic from the normal full shape, cell surface membrane wrinkles were significantly reduced, and no longer extended outward, compared with the normal and full cell morphology of the control cells.

### 2.5. SAB Directly Interacted with β-actin

**Figure 5:**
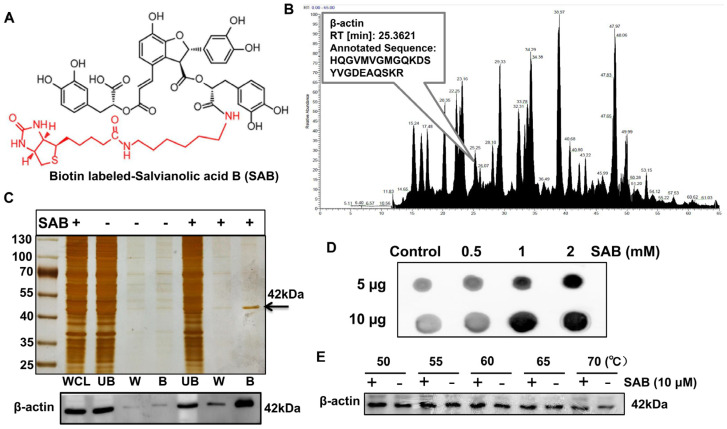
SAB directly interacts with the β-actin protein. (**A**) Chemical structure of the SAB-labeled biotin tag. (**B**) Identification of interacting proteins between SAB and cell lysates using mass spectrometry. The protein with a residence time of 25.3621 was a 42 kDa β-actin protein. (**C**) Silver staining analysis of the enriched protein revealed a distinct protein band at 42 kDa. The band near 42 kDa was detected as β-actin protein by Western blotting. (**D**) Dot blotting of β-actin in solutions containing 0.5, 1, and 2 mm SAB. (**E**) CETSA experiments were conducted at various temperatures with and without 10 μM SAB.

To examine the direct interaction of SAB with β-actin, a biotin tag was added to the structure of SAB (Figure 5A). Biotin-labeled SAB was then used and incubated with HEK293T cell lysates overnight, and then SAB-bound proteins were isolated and enriched using affinity streptavidin magnetic beads. Then, silver staining analysis was performed, and the enriched proteins have obvious protein bands at 42 kDa, and the bands containing 42 kDa were identified by mass spectrometry, among which the protein with a residence time of 25.3621 was β-actin (Figure 5C). The Western blotting assay confirmed that the band around 42 kDa was indeed β-actin protein, which agreed with previous molecular fishing results (Figure 5B). Next, it was also proved by a dot-blotting experiment that SAB could bind to β-actin, and the binding capacity also increased with the increase in SAB concentration (Figure 5D). Finally, by using the CETSA assay in cells, it was found that β-actin protein had a degradation trend at around 50 °C, but the degradation of β-actin protein was reduced after the addition of SAB (Figure 5E). The above results indicate that SAB could directly interact with β-actin protein, and the interaction could make β-actin protein compact and more stable.

### 2.6. The Binding Mode of SAB and β-actin Protein Revealed by Molecular Docking

**Figure 6:**
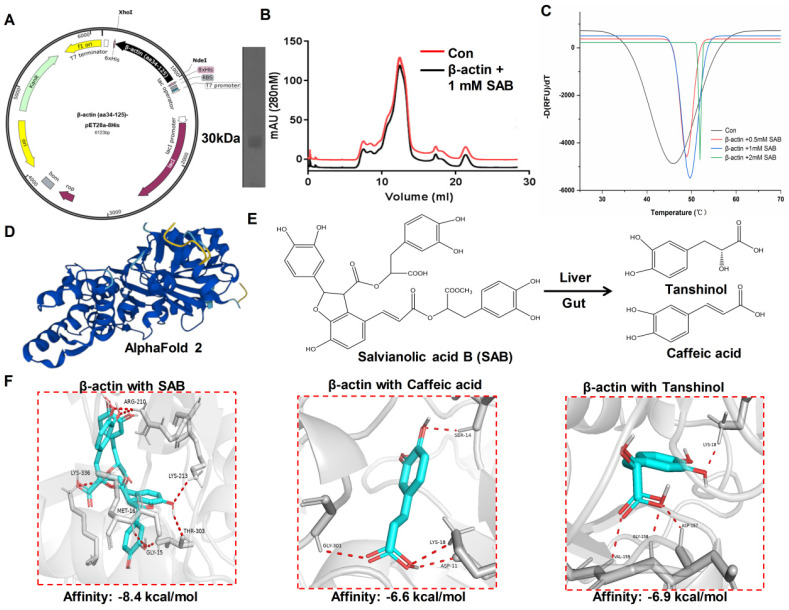
Docked complex of SAB and its products with β-actin. (**A**) Construct map of the pET28a (+) plasmid with the β-actin (aa34-125) gene (left panel), SDS-PAGE gel for β-actin protein purification with a molecular weight of 30 kDa (right panel). (**B**) Size exclusion chromatography analysis. The red and black lines represent the ultraviolet absorption of the control group and β-actin proteins with 1 mM SAB at 280 nM, respectively. (**C**) Thermal stability of β-actin with 0.5, 1, and 2 mM SAB by DSF assay. The Con represents the group of the β-actin protein without compound colored in black. The β-actin protein incubated with 0.5 mM SAB, 1 mM SAB, and 2 mM SAB is colored in red, blue, and green, respectively. (**D**) The protein structure of β-actin, predicted by the AlphaFold 2 online site. The β sheet and the loop structure were marked in blue and yellow, respectively. (**E**) SAB is metabolized by the liver and intestines into smaller molecules of tanshinol and caffeic acid. (**F**) Molecular docking of β-actin with the compound SAB, caffeic acid, and tanshinol. The grey cartoon represents β-actin, and the blue stick represents SAB, caffeic acid, and tanshinol from left to right, respectively. The red dotted line represents the hydrogen bonding interaction between β-actin and SAB, caffeic acid, and tanshinol (distance < 3.5 Å).

The gene fragment corresponding to the 34–125 amino acid sequence of β-actin was cloned into the pET28a (+) vector using cloning technology to construct a complete β-actin-pET28a-8His plasmid, and the *E. coli* expression system was used to overexpress β-actin protein (Figure 6A). Size exclusion chromatography was used to purify and analyze the purity and conformational state of β-actin in solution. After SAB treatment, the protein peak of β-actin was more symmetrical, and the conformation was more stable (Figure 6B). Moreover, the thermal stability of β-actin was higher after SAB treatment compared with the control group (Figure 6C). We downloaded the protein structure of β-actin using the AlphaFold 2 online website. As shown in Figure 6D, the blue-labeled part is the β sheet, and to bind some other important proteins, the yellow fraction is the ring structure. After SAB is metabolized in the intestine and liver, it is broken down into tanshinol and caffeic acid, which have a smaller molecular weight. Studies have shown that both tanshinol and caffeic acid have anti-inflammatory and antibacterial biological activity (Figure 6E). To further determine the binding pattern of SAB to β-actin, we performed molecular docking of SAB, tanshinol, and caffeic acid with β-actin proteins, respectively. Tanshinol and caffeic acid were weak, with affinities of −8.4 kcal/mol, −6.6 kcal/mol, and −6.9 kcal/mol, respectively. Taken together, we inferred that SAB has a stronger interaction with the N terminus of the β-actin protein (Figure 6F).

## 3. Discussion

Melanoma is one of the most frequent malignancies and represents an aggressive form of skin cancer. Advanced stages of melanoma are characterized by a poor prognosis, mainly due to the lack of effective treatments and the development of resistance to chemotherapy [40,41]. Melanoma therapy is characterized by poor efficacy without evidence of significant improvement in prognosis; furthermore, it is limited by several side effects that negatively affect patients’ quality of life [42].

SAB is one of the most abundant and bioactive hydrophilic components in *Salvia miltiorrhiza Bunge*. According to the Chinese Pharmacopoeia, SAB is one of the important reference ingredients in the quality standard of *Salvia miltiorrhiza Bunge* [43]. SAB is primarily used to treat a range of diseases, such as heart disease and neurodegeneration [44,45]. SAB also has therapeutic effects on a variety of cancers, such as lung, breast, oral squamous cell carcinoma, head and neck cancer, hepatocellular carcinoma, and glioma cancer cell lines. SAB can directly bind to ubiquitin-carboxy-terminal hydrolase 2 (USP2) in colon cancer cell RKO, inhibit its deubiquitination activity, enhance the killing activity of T cells in tumor cells, and finally play an anti-tumor role [46]. In addition, SAB can induce autophagy to inhibit the proliferation of colon cancer cells [47]. In this study, we found that SAB has a significant inhibitory effect on the migration of melanoma cell lines, and the inhibitory effect on the growth activity of human A375 cell lines is more obvious, which also suggests that SAB may be a potential drug for the treatment of human melanoma.

EMT is an important transformation process in cancer, which can enhance the flexibility and invasion ability of tumor cells [48,49]. SAB inhibits the growth of TGF-β1-induced A549 human non-small cell lung cancer (NSCLC) cells by inactivating mitogen-activated protein kinase (MAPK) and phosphorylating Smad2/3 [50]. In addition, it can inhibit EMT, cell migration in A549 cells. [51]. Most importantly, we found that SAB directly interacts with β-actin to inhibit EMT transformation and further inhibit melanoma metastasis in this study.

Due to the potential role of EMT in melanoma progression and treatment resistance, several anticancer drugs and small molecules have been developed. These anti-cancer drugs, which are mainly natural products derived from plants, inhibit the EMT process and reduce the progression and invasion of melanoma cells. Therefore, the prevention of tumor metastasis in melanoma by targeting small molecules involved in the EMT mechanism of β-actin may be a landmark advance in the field of tumor metastasis research.

## 4. Material and Methods

### 4.1. Cell Lines

The ATCC was used to obtain the normal skin cells HaCaT, human melanoma A375 cells, and mouse melanoma B16 cells. Then, they were grown in RPMI 1640 mixture with 10% FBS (fetal bovine serum), 100 U/mL penicillin, and 0.1 mg/mL streptomycin, and 5% CO_2_ and a constant incubator temperature of 37 °C with saturation humidity were used to consistently cultivate the cells.

### 4.2. Nature Product

Salvianolic acid B was purchased from Sigma (121521-90-2, St. Louis, MO, USA) and its purity is greater than 95%.

### 4.3. MTT Assay

To evaluate how SAB affected the viability of the HaCaT, A375, and B16 cells, an MTT test was performed. HaCaT, A375, and B16 cells were plated in 96-well plates at a density of 1 × 10^4^ cells/mL. After incubation for 24 h, the cells were treated with various concentrations of SAB (0, 0.390625, 0.78125, 1.5625, 3.125, 6.25, 12.5, 25, 50, and 100 μM) and remained incubated at 37 °C for an additional 24 h. The cells were further treated with 200 μL of MTT (0.5 mg/mL) for an additional 4 h at 37 °C after the initial incubation. After discarding the supernatant, 150 μL of DMSO was added to each well, and each was then given 10 min of incubation. Finally, a microplate reader was used to measure the absorption of each well at 490 nm.

### 4.4. EdU Assay

HaCaT, A375, and B16 cell proliferation was assessed by an EdU experiment in accordance with the protocols. HaCaT, A375, and B16 cells were seeded into 96-well plates and treated with various concentrations of SAB (0, 12.5, 25, and 50 μM) for 24 h. The cells were then exposed to 50 μM of EdU for 2 h at 37 °C. The cells were then preserved with 4% formaldehyde, permeabilized with 0.3% Triton X-100, incubated with click reaction solution, and finally stained with Hoechst 33342 dye. Thereafter, using a fluorescence microscope, the cell nuclei were stained with Hoechst 33342. (Leica, Wetzlar, Germany). Based on the green color of positive cells, the cell proliferation index was computed as the ratio of EdU to Hoechst 33342.

### 4.5. Wound Healing Assay

For the wound healing assay, 5 × 10^5^ cells/well (three replicates per group) were plated into a 6-well plate and incubated until confluence. To remove the detached cells, the monolayer was scraped with a tip and then rinsed in PBS. The cells were then grown in mixed medium with SAB (0, 12.5, 25, 50 μM) added. A375 and B16 cells were photographed at 0, 12, 24 and 48 h.

### 4.6. Western Blot Analysis

After treatment, RIPA buffer mixed with a protease inhibitor cocktail was used to lyse both A375 and B16 cells. Following the manufacturer’s instructions, protein concentrations were measured using a BCA (bicinchoninic acid) measurement kit. Western blotting was performed using primary antibodies against N-cadherin, E-cadherin, vimentin, and β-actin (1:1000). Image J 1.54h was used to calculate the band intensities’ densitometry.

### 4.7. Immunofluorescence

A375 and B16 cells were seeded on confocal dishes and subjected to SAB (0, 12.5, 25, and 50 μM) for 24 h in order to perform immunofluorescence labeling on the cells. The cells were permeabilized with 0.3% TritonX-100, fixed in 4% paraformaldehyde, and blocked with bovine serum albumin at the end of the incubation. Then, the cells were incubated with a β-actin (1: 200) antibody at 4 °C overnight and then stained with a secondary fluorescent antibody (1: 1000; Alexa Fluor 488, Abcam Inc., Cambridge, MA, USA). The cells were then given another 30 min of DAPI (5 μg/mL) incubation. A Leica TCS SP8 confocal fluorescence microscope was used to capture fluorescent signals (Leica, Germany).

### 4.8. Biotin Labeled SAB and Molecular Fishing

SAB-NHS-biotin was synthesized using tricarballylic acid, *O*-benzylhydroxylamine hydrochloride, SAB, and biotin using standard chemical procedures. Then, the products were deprotected and activated, and biotin was linked to the activated NHS ester. Biotin-labeled SAB was separated by HPLC and identified by NMR. Then, SAB was labeled with biotin and then incubated with HEK293T cell lysate for 2 h. The protein bound to SAB was further isolated with streptavidin magnetic beads (Solarbio, Beijing, China). The binding proteins were eluted with SDS-loaded dyes, analyzed by SDS-PAGE, and then visualized by silver stain. Then, SAB-binding proteins were used for mass spectrometry.

### 4.9. Clone of β-actin into the pET-28a-psp Expression Vector

Human β-actin (NM_001101) expression targeting sequence (aa34-125) was obtained from GeneCopoeia. The pET-28a plasmid is linearized by double digestion of *Xho*I and *Nde*I restriction endonuclease enzymes; the amplified PCR product is purified with the DNA gel extraction kit 50-Prep, it was purchased from Thermo Scientific (Thermo Scientific, Waltham, MA, USA); and the amplified PCR product is ligated and recombined with the linearized pET-28a vector by T4 ligase. Next, the recombinant plasmid is expanded with DH5a cells and diffused onto LB agar plates containing 34 μg/mL kanamycin. Finally, colony PCR validation and sequencing sequence alignment were performed.

### 4.10. Expression and Purification of Recombinant Human β-actin

Expression and purification of β-actin (aa34-125) were performed with standard procedures. Briefly, *E. coli* Rosetta (DE3) cells transformed with the β-actin plasmid were grown in terrific broth supplemented with 34 mg/mL kanamycin and 35 mg/mL chloramphenicol at 37 °C. The cells were grown at 37 °C with shaking at 180 rpm until OD600 reached 0.6–0.8, and then protein expression was induced with 0.2 mM β-D-1-thiogalactopyranoside (IPTG) at 25 °C for 12 h. Then, the strains were collected and resuspended in 40 mM Tris-HCl, pH 8.0, 250 mM sodium chloride, 1 mM β-mercaptoethanol, and 1 mM PMSF, and crushed with a low-temperature overpressure battery. The resulting lysate was centrifuged at 14,000 rpm for 60 min to obtain the supernatant. First, the protein of interest was purified using buffer A (250 mM NaCl, 40 mM Tris-HCl, 10 mM imidazole) in combination with Ni^2+^-nitritriotriacetic acid (Ni-NTA) agarose resin (GE Medical, Uppsala, Sweden) in an affinity chromatography step, followed by elution of the protein of interest with buffer B (250 mM NaCl, 40 mM Tris-HCl, 250 mM imidazole). Second, secondary purification was performed on a 1 mL HisTrap HP column (GE Healthcare, Uppsala, Sweden). Third, 10/300 GL (GE Healthcare) was added to the Superdex 200 (Cytiva, Vancouver, BC, Canada) to isolate the purity protein of interest. Finally, the highest purity protein of interest was stored at −80 °C.

### 4.11. Protein Thermal Shift Assay

The purified protein sample was diluted to 5.0 mg/mL and incubated with 0.5 mM, 1 mM, and 2 mM SAB for 30 min on ice, respectively. SYPRO Orange Protein Gel Stain 5000× (Thermo Fisher Scientific, Waltham, MA, USA) is diluted to 200× with protein buffer. The reaction system with a total volume of 20 μL consists of 30 μg containing protein or protein with SAB mixture, 2.5 μL of 200× SYPRO. Heat the mixture from 25 °C to 99 °C at a rate of 0.2 °C/min. Detection was performed with a real-time PCR instrument (7500 Fast, Thermo Fisher Scientific, Waltham, MA, USA). Finally, all protein thermal shift maps were made using Origin 2021.

### 4.12. Dot Blot Assay

The A375 cells were lysed with 1 × RIPA Buffer for 1 h and then centrifuged for 1 h, followed by quantification of the extracted total protein. Whole cell lysate (WCL) and 0.5 mM, 1 mM, and 2 mM SAB were incubated on ice for 1.5 h, respectively. The proteins were quantified, spotted on polyvinylidene fluoride (PVDF) membranes, naturally air dried, and blocked with 5% skim milk powder in TBST for 1 h. Membranes were incubated with a β-actin antibody overnight, washed, incubated with a secondary antibody, and exposed to ECL for detection.

### 4.13. Molecular Docking

Receptor β-actin was obtained from AlphaFold 2 in the form of PDB. Autodock software 4.2.6 was used for molecular docking. The protein was prepared by assigning bond orders, adding hydrogens, setting proper ionization states of residues, and capping. The protein was then refined with H-bond assignment. The ligands were prepared in ligprep with the following parameters: OPLS3e, not changing ionization states and generating tautomers and stereoisomers (creating all combinations of specified chiralities and determining chiralities from 3D structure) with at most 10 ligands to be made. Finally, the regions with the most hydrogen bond interactions and closest to each other were selected for analysis, and the latest optimal poses for SAB, tanshinol, and caffeic acid were selected using the docking score.

### 4.14. Statistical Analysis

The results were presented as the mean ± SD from at least three independent experiments. Data analysis was performed using a two-tailed unpaired Student’s *t* test for two-group comparisons. Multiple comparisons were carried out by one-way analysis of variance (ANOVA), followed by Dunnett’s test with GraphPad Prism 8 (GraphPad Software, Inc., La Jolla, CA, USA). *p* < 0.05 was considered statistically significant.

## 5. Conclusions

Taken together, SAB could significantly suppress the migration of melanoma cells via direct binding with β-actin, suggesting that SAB could be a helpful supplement that may enhance chemotherapeutic outcomes and benefit melanoma patients.

## Data Availability

The data that support the findings of this study are available from the corresponding author, upon reasonable request.
